# Treatment of ongoing autoimmune encephalomyelitis with activated B-cell progenitors maturing into regulatory B cells

**DOI:** 10.1038/ncomms12134

**Published:** 2016-07-11

**Authors:** Sarantis Korniotis, Christophe Gras, Hélène Letscher, Ruddy Montandon, Jérôme Mégret, Stefanie Siegert, Sophie Ezine, Padraic G. Fallon, Sanjiv A. Luther, Simon Fillatreau, Flora Zavala

**Affiliations:** 1 Institut Necker Enfants Malades, Immunology, Infectiology and Haematology Department, Inserm U1151, CNRS UMR 8253, 14 rue Maria Helena Vieira da Silva, CS 61431, Paris 75014, France; 2 Université Paris Descartes, Sorbonne Paris Cité, Faculté de Médecine Site Necker, 14 rue Maria Helena Vieira da Silva, CS 61431, Paris 75014, France; 3 Structure Fédérative de Recherche Necker, INSERM US 24, CNRS UMS 3633, Paris 75014, France; 4 Department of Biochemistry, University of Lausanne, Epalinges 1066, Switzerland; 5 Department of Medicine, Trinity Biomedical Sciences Institute, Trinity College Dublin, Dublin 2, Ireland; 6 Assistance Publique—Hopitaux de Paris (AP-HP), Hopital Necker Enfants Malades, Paris 75015, France; 7 Deutsches Rheuma-Forschungszentrum, a Leibniz Institute, Chariteplatz 1, Berlin 10117, Germany

## Abstract

The influence of signals perceived by immature B cells during their development in bone marrow on their subsequent functions as mature cells are poorly defined. Here, we show that bone marrow cells transiently stimulated *in vivo* or *in vitro* through the Toll-like receptor 9 generate proB cells (CpG-proBs) that interrupt experimental autoimmune encephalomyelitis (EAE) when transferred at the onset of clinical symptoms. Protection requires differentiation of CpG-proBs into mature B cells that home to reactive lymph nodes, where they trap T cells by releasing the CCR7 ligand, CCL19, and to inflamed central nervous system, where they locally limit immunopathogenesis through interleukin-10 production, thereby cooperatively inhibiting ongoing EAE. These data demonstrate that a transient inflammation at the environment, where proB cells develop, is sufficient to confer regulatory functions onto their mature B-cell progeny. In addition, these properties of CpG-proBs open interesting perspectives for cell therapy of autoimmune diseases.

## Abstract (web summary)

Evidence of how functional Bregs develop *in vivo* has been lacking. Here the authors show that proB cells exposed *in vivo* to CpG differentiate into distinct Breg subsets that inhibit autoimmunity by arresting T cells in the lymph nodes via CCL19 and by producing IL-10 at the site of immunopathology.

## Graphical abstract

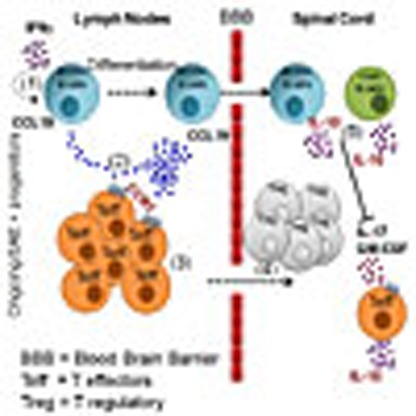

B lymphocytes exert complex functions in autoimmune diseases. On the one hand they can promote these diseases, as shown by the beneficial effects of B-cell depletion therapies in rheumatoid arthritis or multiple sclerosis (MS)^1^,^2^,^3^. On the other hand, their negative regulatory functions can provide protection, as initially shown in models of ulcerative colitis^4^, experimental autoimmune encephalomyelitis (EAE)^5^ and collagen-induced arthritis^6^. More precisely, mice with an interleukin (IL)-10 deficiency restricted to B cells developed a severe chronic form of EAE, while those harbouring wild-type (WT) B cells rapidly recovered from disease^5^. The unique capacity of B cells to reduce the severity of autoimmune diseases through provision of IL-10 has kindled enormous interest in the identification of the responsible B-cell sub-populations, and the signals controlling their expression of suppressive functions. Several B-cell subsets can produce IL-10 on stimulation *in vitro*, and protect recipient mice from autoimmune disease on adoptive transfer. These include B10 cells with a CD5^+^CD1d^high^ phenotype, transitional type 2 (T2)-marginal zone precursors, and CD5^+^ B-1 cells (refs 7, 8, 9). Moreover, analysis of the cells producing IL-10 in a suppressive manner *in vivo* identified CD138^hi^ plasma cells residing either in spleen^10^ or LN^11^ as major IL-10 producers during EAE. In addition, IL-35 (ref. 10) and PD-L1 (ref. 12) were recently shown to mediate protection against EAE displayed by B regulatory cells.

Toll-like receptor (TLR) agonists are particularly important in this context because of their unique capacity to induce IL-10 expression in mature naive B cells, and the requirement for intrinsic TLR signalling in B cells for recovery from EAE^13^. Similarly, CD5^+^CD1d^high^ B cells depend on activation by TLR-4 or -9 agonists to produce IL-10 *in vitro*^14^. In addition to intrinsic TLR signalling, signals provided to B cells through the BCR or CD40 are also necessary to achieve a fully protective B-cell-mediated regulation^5^,^15^,^16^,^17^,^18^,^19^.

Human B cells can also produce IL-10, and evidence is accumulating that they can subsequently behave as negative regulators of immunity. Duddy *et al.*^20^ found that B cells from MS patients produce lower amounts of IL-10 on activation than their counterparts from healthy individuals, suggesting that MS is facilitated by a defect in this regulatory circuit. This defective IL-10 production was subsequently confirmed by Correale and Farez^21^ who observed that helminth infections could restore normal IL-10 production by B cells in this disease, which correlated with an improvement of the disease course compared with non-infected patients. These data suggest that promoting B-cell-mediated regulation might help to reduce MS progression. Of note, some MS treatments, such as interferon (IFN)-β^22^ or glatiramer acetate^23^,^24^ have been shown to enhance IL-10 secretion by B cells.

Although our knowledge of the peripheral B-cell subsets implicated in immune regulation, and the signals controlling their regulatory activity has greatly improved, we still have little data on whether B-cell development in bone marrow (BM) might influence the subsequent capacity of mature B cells to negatively regulate immunity in periphery. We recently gained some evidence of this by showing that proB cells emerging from BM cell cultures transiently stimulated with the TLR-9 agonist CpG prevented type 1 diabetes in NOD mice on adoptive transfer^25^. These cells, which we termed as CpG-proB cells, differentiated into various more mature B-cell subsets in recipient mice. We could demonstrate that they provided protection by reducing pathogenic IL-21 secretion by T cells, and by promoting apoptosis of T-effector cells^25^. However, it was difficult to further analyse how the administered CpG-proBs protected recipient mice from disease in this model due to the lack of mutant mouse strains on the NOD background. We therefore addressed the question, whether a similar subset emerged in the C57BL/6 mouse strain. To this end, we used the myelin oligodendrocyte glycoprotein (MOG)_35–55_-induced EAE model in which B-cell-mediated regulation has been extensively studied. So far described regulatory B-cell subsets only acted at the initiation phase of the disease, except PD-L1^hi^ mature splenic B cells of which 1 million cells injected at day 7 after immunization provided protection against EAE^12^, albeit milder than when injected at day 0. However, we find that a single injection of only 60,000 CpG-proB cells at the onset of EAE clinical signs markedly reduces disease progression in recipient mice. In contrast, adoptive transfer of control non-stimulated proBs does not influence the disease course in recipient mice. This suppression requires the differentiation of the administered CpG-proB cells into mature B cells in the periphery and in the central nervous system (CNS). Collectively, these results shed light on the importance of the BCR-independent signals that B-cell progenitors receive in the BM environment to develop into mature B cells with regulatory rather than inflammatory functions. In addition, these properties of CpG-proBs open interesting perspectives for cell therapy of autoimmune diseases.

## Results

### ProB cells exposed to CpG protect against ongoing EAE

**Figure 1:**
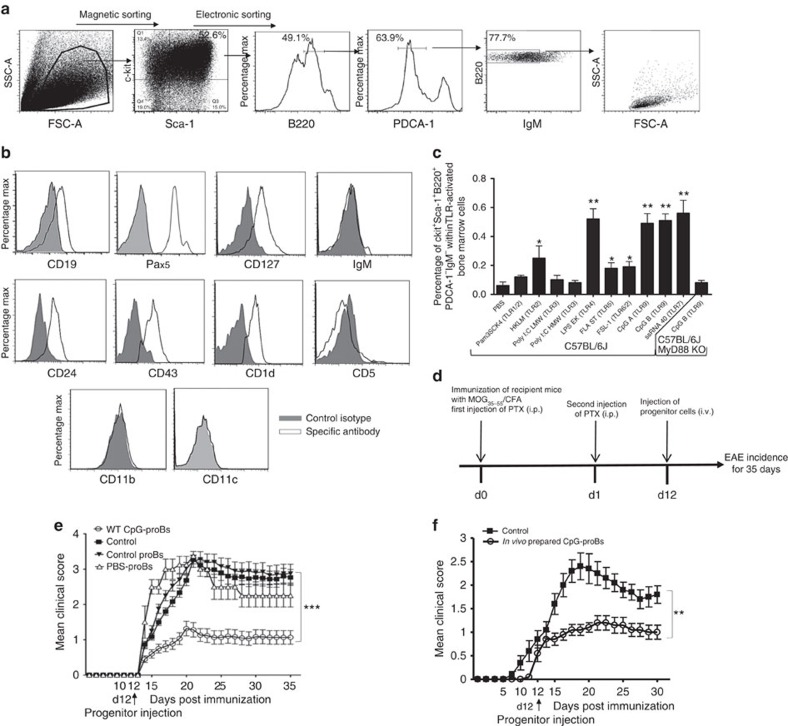
Phenotypic analysis of CpG-induced c-kit^+^Sca-1^+^B220^+^PDCA-1^−^IgM^−^ BM cells and assessment of disease protection against ongoing EAE. (**a**) BM cells incubated for 18 h with CpG-B (1 μg ml^−1^), were magnetically selected for c-kit^+^ cells, further labelled for Sca-1, B220, PDCA-1 and IgM and electronically sorted into small-size (FSC^low^SSC^low^) c-kit^+^Sca-1^+^B220^+^PDCA-1^−^IgM^−^ cells. (**b**) Flow cytometry analysis of indicated B-cell markers expression by CpG-proB cells after cell-sorting as in **a**. (**a**,**b**) Cells were stained with specific antibodies (open histograms) or isotype controls (filled histograms). (**c**) Frequency of c-kit^+^Sca-1^+^B220^+^PDCA-1^−^IgM^−^ cells emerging among BM cells after 18 h of incubation with different TLR agonists. CpG-B was tested in BM cell cultures of both WT and MyD88^−/−^ C57BL/6J mice. Results are expressed as means±s.e.m. from three experiments. *P<0.05, **P<0.005 when comparing stimulated and unstimulated BM cells, using non-parametric Mann–Whitney's *t*-test. (**d**) Experimental protocol for MOG_35–55_ EAE disease induction and intravenous progenitor cell transfer (60,000 cells per mouse) at day 12 post-immunization. (**e**,**f**) EAE clinical scores (mean±s.e.m.) over 35 days of the indicated groups of mice. (**e**) *n*=30 mice per group, except for PBS-ProB-recipient group in which *n*=4 mice. (**f**) *n*=10 mice per group; ***P<0.001 when comparing control mice injected with PBS and recipients of WT CpG-proBs by two-way repeated measures ANOVA test. **P<0.005, between mice injected with *in vivo* prepared CpG-proBs and other groups, non significant between all other groups.

We previously found that on short-term incubation with CpG-1668 (CpG-B), BM cells from NOD mice gave rise to a population of pro-B cells that protected recipient mice from diabetes on adoptive transfer^25^. To assess whether a similar process took place in a strain of mice which are more amenable to genetic studies, we evaluated the effect of CpG-B on BM cells from C57BL/6J mice. This treatment led to emergence of cells expressing a c-kit^+^Sca-1^+^B220^+^IgM^−^ phenotype, and heterogeneous in terms of PDCA-1 expression (Fig. 1a and Supplementary Fig. 1). A similar BM population was observed *in vivo* in mice after i.p injection of CpG-B, validating the use of *in vitro* cultures (Supplementary Fig. 2). The bright B220^+^ cells are gated out since they correspond to the more mature B cells contaminating the c-kit^+^ magnetically sorted cells. Moreover, since TLR-9 stimulation has been shown to promote deviation of hematopoiesis away from the B-cell lineage towards the PDCA-1^+^ plasmacytoid dendritic cell lineage^26^, B-cell precursors were further sorted by excluding the PDCA-1^+^ fraction (Fig. 1a). The resulting PDCA-1^−^ population was closely related to the pro-B cell stage of differentiation, being CD19^+^CD24^+^IgM^−^CD11b^−^CD11c^−^, as well as expressing the IL-7Rα chain (CD127), CD43 and the transcription factor Pax5 (Fig. 1b and Supplementary Fig. 3a) characterizing B-cell lineage commitment. They all expressed CD1d, but were negative for CD5 (Fig. 1b). It is noteworthy that this effect was not restricted to TLR-9 agonists, because agonists of TLR-2, -4, -5, -6 and -7 induced development of a similar population, unlike agonists of TLR-1 and -3 (Fig. 1c). As expected, these cells did not appear in BM cell cultures from MyD88-deficient mice after incubation with CpG-B (Fig. 1c). Collectively, these data suggest that TLR agonists induce *in vitro* and *in vivo* the formation of a unique population of proB cells in BM from C57BL/6 mice, as previously found in NOD mice^25^.

We next examined whether these cells could protect recipient mice from EAE on adoptive transfer. Remarkably, a single injection of only 60,000 CpG-proBs (Fig. 1d) isolated either from *in vitro* BM cell culture activated with CpG (Fig. 1e, Table 1) or from BM of CpG-injected donors (Fig. 1f) to mice at the time of EAE onset (d12 after immunization) resulted in a marked attenuation of the disease course, relative to control mice that received only phosphate-buffered saline (PBS). Conversely, neither control pro-B cells isolated using their typical markers CD24 and CD43, from fresh non-stimulated BM, as c-kit^+^Sca1^−^B220^+^CD24^hi^CD43^hi^ cells (Fig. 1e and Supplementary Fig. 3b), nor cells detected within BM culture with PBS over 18 h exhibiting the same phenotype as CpG-proB cells but at a 6–10-fold less frequency, termed PBS-proBs, had any effect in recipient mice (Fig. 1e). Therefore transient *in vitro* and *in vivo* TLR-9-dependent activation within the BM confers protective properties onto proB cells against ongoing EAE. A dose–response study (Supplementary Fig. 4) showed that injection of 60,000 CpG-proBs was optimal for protection against EAE. Neither 25,000 nor 90,000 injected cells provided significant protection, the latter possibly resulting from cell aggregation or, alternatively, from hindrance in access of cells into the hematopoietic niche of non-irradiated recipients.

### CpG-proB cells modify immune cell response and distribution

**Figure 2:**
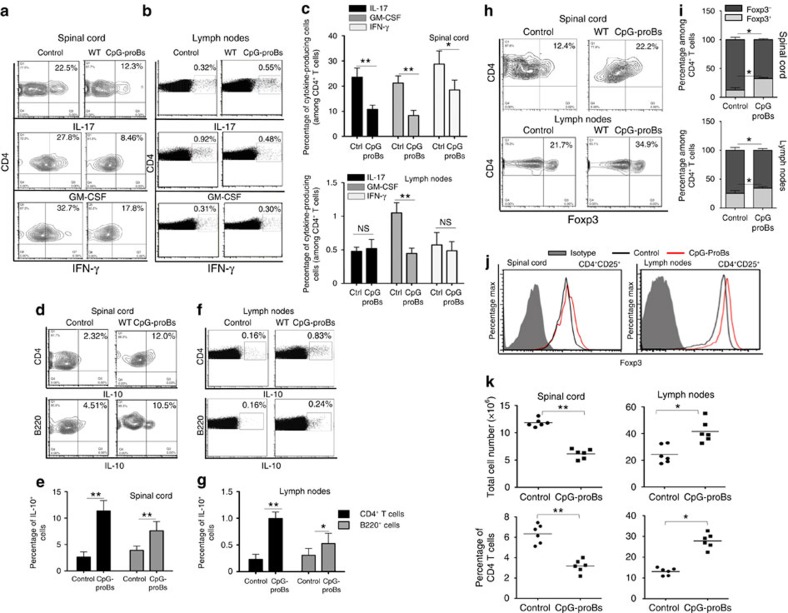
Protective effects of CpG-proBs at the peak of disease. (**a**–**c**) Frequency at day 21 of disease of CD4^+^ cells producing IL-17, GM-CSF, IFN-γ in spinal cord in a representative experiment (**a**) and in four experiments (**c**), and in reactive (cervical, axillary and inguinal) LN (**b**,**c**) prepared as described in the ‘Methods' section. (**d**–**g**) Production of IL-10 by CD4^+^ T cells and B220^+^ B cells from spinal cord (**d**,**e**) or reactive LN (**f**,**g**) of control or CpG-proB-recipient mice in a representative experiment (**d**,**f**) and in three experiments (**e**,**g**) in which values are expressed as mean±s.e.m. (**h**) Frequency within gated CD4^+^ cells of cells with nuclear expression of the transcription factor Foxp3 in spinal cord and reactive LN. (**i**,**j**) Percentages of Foxp3^+^ and Foxp3^−^ CD4^+^CD25^+^ T cells from spinal cord and reactive LN out of four experiments (**i**), also shown in **j** from a representative flow cytometry experiment in control mice (black line) and CpG-proB recipients (red line). Cells were stained for Foxp3 (open histograms) versus isotype control (filled histograms). (**k**) Total cell counts and percentage of CD4^+^ T cells within spinal cord and reactive LN of control or CpG-proB-recipient mice at the peak of the disease. *P<0.05, **P<0.005, when comparing CpG-proB-injected mice and other groups. *n*=6 mice per group (Students' *t*-test).

To characterize the protective effect of the injected progenitors on encephalitogenic T cells, we analysed the expression of pro-inflammatory cytokines by CD4^+^ T cells in treated mice at the peak of the disease (d18–d21). In spinal cord, which is a major target organ in EAE, mice treated with CpG-proB cells had reduced numbers of CD4^+^ T cells producing all pathogenic cytokines tested, namely GM-CSF, IL-17 and IFN-γ (Fig. 2a,c). In LN, production of GM-CSF by CD4^+^ T cells was reduced, whereas expression of IL-17 and IFN-γ were not affected in treated mice compared with controls (Fig. 2b,c). In addition, production of the anti-inflammatory cytokine IL-10 by CD4^+^ T and B220^+^ B cells was enhanced in treated mice in both tissues (Fig. 2d–g), which was paralleled by an increased frequency of CD4^+^Foxp3^+^ regulatory T cells (Fig. 2h,i), showing a modestly enhanced Foxp3 expression (Fig. 2j).

To further delineate how the transferred CpG-proBs protected from EAE, we analysed immune cell distribution in recipient mice, in comparison with control mice with EAE. Remarkably, mice treated with CpG-proB cells displayed increased cell numbers and particularly CD4^+^ T cells in LN, while having markedly fewer total and CD4^+^ T cells in spinal cord compared with controls (Fig. 2k).

In sum, these data show that the administered B cells impact on the disease process both at the level of the CNS and LN.

### CpG-proB cells migrate and differentiate into mature Bregs

**Figure 3:**
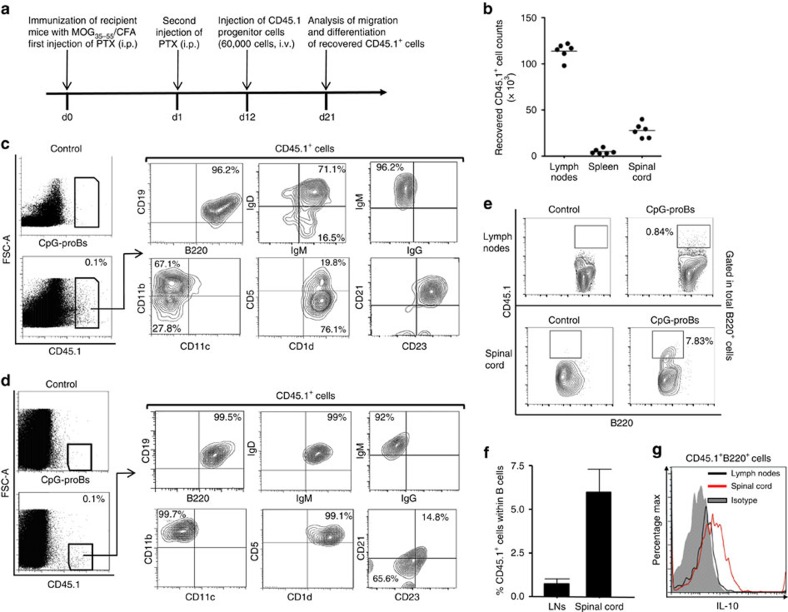
Phenotypic characterization of CD45.1^+^ cells recovered from reactive LNs and spinal cord. (**a**) Experimental protocol: EAE disease was induced in CD45.2 C57BL/6J mice which then were injected at day 12 of the disease with CpG-proB cells, prepared from the BM of congenic CD45.1^**+**^ C57BL/6J and stimulated with CpG-B for 18 h. The recovery (**b**) and differentiation status (**c**–**e**) of CD45.1^+^ injected cells were analysed at day 21. (**b**) Absolute CD45.1^+^ cell counts recovered within reactive LN, spleen and spinal cord of injected mice at day 21 after immunization, that is, at the peak of the disease, *n*=6 mice per group. (**c**) Flow cytometry analysis of the frequency of CD45.1^+^ cells recovered in reactive LN at day 21, and of the frequency of gated CD45.1^+^ cells expressing the B-cell markers B220, CD19, IgM, IgD, IgG, as well as the markers CD5 and CD1d, CD11b, and CD11c, CD21 and CD23. (**d**) Flow cytometry analysis of the frequency of the CD45.1^+^ progeny of CpG-proB cell recipients, recovered within the spinal cord of CpG-proB-injected mice at day 21 of the disease, expressing B220, CD19, IgM, IgD, IgG, CD5, CD1d, and CD11b, CD11c, CD21 and CD23. (**e**,**f**) Frequency of CD45.1^+^ cells derived from injected CpG-proBs gated within total B220^+^ cells in a representative experiment (**e**) and as mean±s.e.m. of three determinations (**f**). (**g**) Frequency of IL-10-producing CD45.1^+^ cells in spinal cord and reactive LN, determined by intracellular flow cytometry as shown in the ‘Methods' section.

To trace the migration and follow the differentiation of the administered CpG-proB immature cells, we injected 60,000 CpG-proB cells prepared from BM of congenic CD45.1 mice into CD45.2 recipient mice at day 12 after EAE induction (Fig. 3a). On day 9 after transfer, CD45.1^+^ cells were mainly found in reactive, cervical, axillary and inguinal, but not mesenteric, lymph nodes (LNs; 113,900±3,500 cells, mean±s.e.m.), and spinal cord (27,880±3,218 cells), with only few cells present in spleen (5,000±1,098 cells; Fig. 3b). It is noteworthy that the total number of donor-derived cells was about threefold higher in recipients than the number of injected cells, indicating that they expanded *in vivo*. Using different B-cell and other lineage markers, we assessed that the transferred CpG-proBs differentiated exclusively into the B-cell lineage (Fig. 3c,d). In LN, the recovered CD45.1^+^ cells displayed a B220^+^CD19^+^IgM^+^IgD^+^IgG^−^CD1d^+^CD21^+^CD23^hi^ phenotype typical of transitional B cells (Fig. 3c). This population was, however, heterogeneous, comprising about 70% of CD11b^+^CD11c^−^, and 20% of CD5^+^ cells (Fig. 3c). In spinal cord, donor-derived B cells were comparable to their LN counterparts in their B220^+^CD19^+^IgM^+^IgD^+^IgG^−^ phenotype (Fig. 3d) but with lower levels of CD21 and CD23 than in LNs and exclusively CD1d^+^CD5^+^CD11b^+^, therefore similar to B10 cells. While the CD45.1^+^ B-cell progeny of injected progenitors represented only 0.77±0.14% of B cells in the LNs, they represented nearly eightfold more, that is, 5.97±1.32% of B cells, in the spinal cord (Fig. 3e,f). Remarkably, CD45.1^+^ B cells produced the anti-inflammatory cytokine IL-10 in spinal cord, but not in LN (Fig. 3g).

These data demonstrate that the transferred CpG-proB cells give rise to more mature B cells that accumulate in reactive—cervical and inguinal—LN and inflamed CNS during EAE.

**Figure 4:**
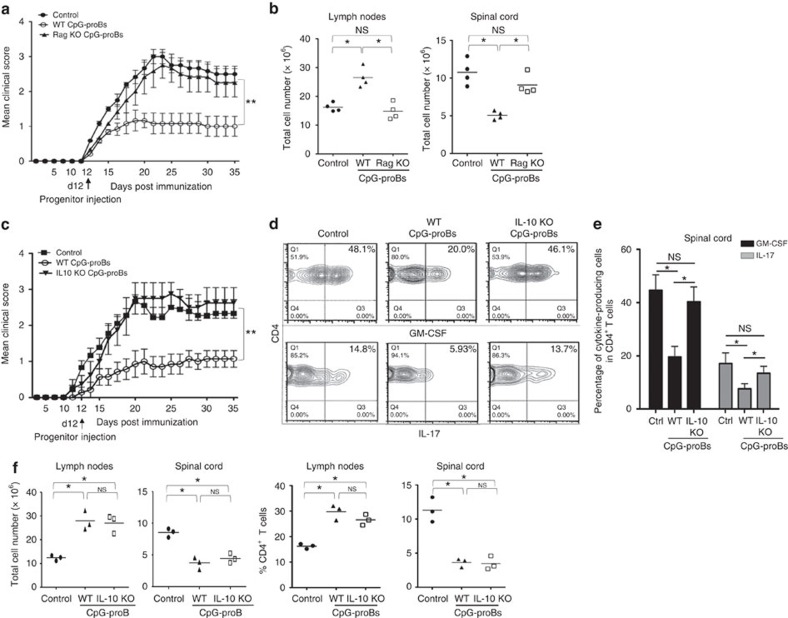
Role of maturation and IL-10 production capacities of CpG-proBs in the protection against EAE. (**a**) EAE clinical scores (mean±s.e.m.) over 35 days of the indicated groups of mice. *n*=30 mice per group for control mice and for recipients of WT CpG-proBs, *n*=8 mice per group for recipients of *Rag2*^−/−^-derived CpG-proBs; ***P<0.001 when comparing control mice injected with PBS and recipients of WT CpG-proBs by two-way repeated measures ANOVA test. **P<0.005, between mice injected with WT CpG-proBs and other groups, non significant between all other groups. (**b**) Total cell number found in LNs and spinal cord of control mice and recipients of WT or Rag2^−/−^ CpG-proBs (*n*=4 mice per group). (**c**) EAE clinical scores (mean±s.e.m.) in control mice or recipients of CpG-proB cells derived from the BM of WT or IL-10^−/−^ mice. *n*=8 mice per group. **P<0.005, as assessed by two-way repeated measures ANOVA test, only between mice injected with WT CpG-proBs and other groups, non significant between all other groups. (**d**,**e**) Frequency of CD4^+^ T cells producing GM-CSF and IL-17 in the spinal cord of control mice with EAE, recipients of WT- or of IL-10^−/−^- CpG-proBs, shown in a representative experiment (**d**), and in three experiments (**e**) in which values are expressed as mean±s.e.m. (**f**) Total cell counts and frequency of CD4^+^ T cells recovered from reactive LN and spinal cord of control mice with EAE, or from recipients of WT- and IL-10^−/−^ CpG-proBs (*n*=3 mice per group). *P<0.05 (Students'*t*-test).

To assess whether such differentiation of CpG-proB cells into more mature B cells was required for protection against EAE, CpG-proB cells from CD45.1^+^*Rag2*^−/−^ mice (prepared as in Supplementary Fig. 5) whose differentiation into mature B lymphocytes is blocked, were adoptively transferred. They had no effect on the disease course (Fig. 4a). Injected cells from CD45.1^+^*Rag2*^−*/*−^ donors could not be found in LNs or spinal cord at 3, 5 or 7 days after injection, in contrast to CpG-proB cells from WT donors, suggesting that their reduced lifespan as progenitors hampered them to confer protection against EAE. Therefore, transient treatment of BM cells with TLR-9 agonist generates proB cells that differentiate into more mature B cells with protective function in EAE. Remarkably, CpG-proB cells from *Rag2*^−/−^ mice had no effect on cell numbers in LN and CNS (Fig. 4b), suggesting that this alteration of cell distribution directly contributed to the beneficial effect afforded by CpG-proB cells.

### Roles of IL-10 production by donor-derived B cells

Since IL-10 is generally a key mediator of B-cell-mediated regulation, we assessed its importance for the suppressive function of CpG-proB cells. IL-10 was necessary for the protective function of CpG-proB cells, because mice treated with IL-10-deficient CpG-proBs (prepared as in Supplementary Fig. 5) were not protected against disease (Fig. 4c), and production of pathogenic cytokines by CD4^+^ T cells in spinal cord was not decreased (Fig. 4d,e). However, IL-10-deficient CpG-proBs still promoted CD4^+^ T cell accumulation in the reactive LN and reduced their numbers in spinal cord (Fig. 4f) suggesting the involvement of an additional mechanism in this latter effect.

Altogether, these data show that an efficient control of disease by CpG-proBs requires IL-10 production by their mature B-cell progeny. IL-10 produced by these cells is important to reduce inflammation locally in spinal cord, while trapping of immune cells in reactive LN is achieved via an IL-10-independent mechanism.

### Role of IFN-γ in LN entrapment of T cells by CpG-proBs

**Figure 5:**
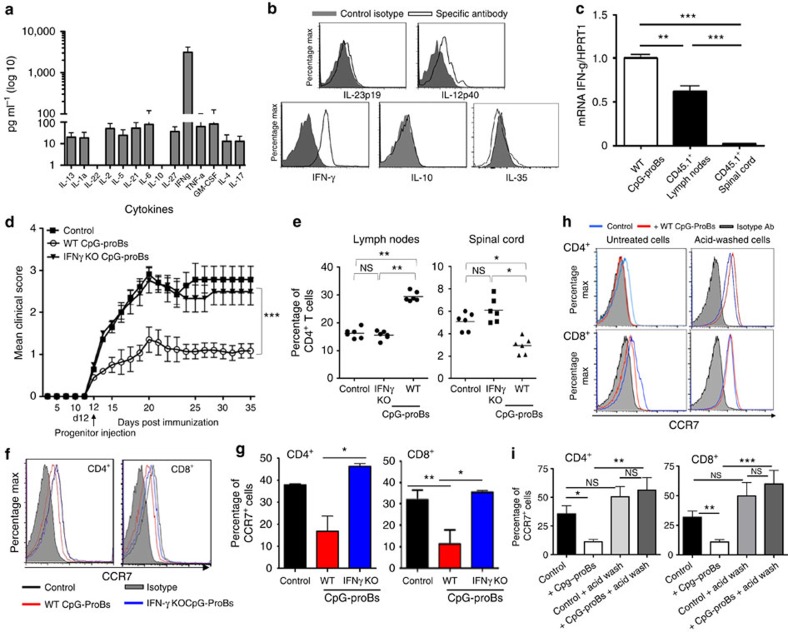
Role of IFN-γ in the protective properties of CpG-proBs. (**a**) Cytokine level determination by multiplex ELISA for Th1/Th2/Th17 cytokines and GM-CSF (expressed in pg ml^−1^) in supernatants of CpG-proB cells (50,000 cells per 200 μl) stimulated for 5 h with PMA+ionomycin. (**b**) Constitutive cytokine production by WT CpG-proB cells. Immediately after FACS sorting, WT CpG-proB cells were stained for intracytoplasmic production of cytokines IL-23p19, IL-12p40, IFN-γ, IL-10 and IL-35 using specific antibodies (open histograms) or isotype controls (filled histograms). (**c**) IFN-γ mRNA levels relative to HPRT1, as determined by qRT–PCR in WT CpG-proBs, their CD45.1^+^ progeny recovered from reactive LN or from spinal cord. Values are expressed as mean±s.e.m. of three experiments (**d**) EAE clinical score (mean±s.e.m.) in control mice (*n*=30) and mice injected at day 12 of the disease with 60,000 WT (*n*=30) or IFN-γ^−/−^ CpG-proB cells (*n*=20). Data are pooled from two independent experiments. ***P<0.001 when comparing disease scores for control mice with those obtained after treatment with WT CpG-proB cells by two-way repeated measures ANOVA test. (**e**) Frequency of CD4^+^ T cells found in reactive LN and spinal cord of control mice versus mice transferred WT or IFN-γ^−/−^ CpG-proB cells, *n*=6 mice per group, *P<0.05 (Students'*t*-test). (**f**–**g**) Expression of CCR7 analysed by flow cytometry in CD4^+^ and CD8^+^ cells from reactive LN of control mice with EAE versus mice transferred either WT or IFN-γ^−/−^ CpG-proB cells. Shown are a representative experiment (**f**) and the mean±s.e.m. of the percentages of CCR7-positive CD4^+^ and CD8^+^ cells out of four experiments (**g**). (**h**,**i**) Expression of CCR7 analysed by flow cytometry either in untreated cells or after an acid wash and shown in a representative experiment (**h**) and as the mean±s.e.m. of the percentages of CCR7-positive CD4^+^ and CD8^+^ cells out of three experiments (**i**). *P<0.05, **P<0.005 (Students' *t*-test).

To identify the IL-10-independent mechanism involved in the accumulation of immune cells in the reactive LN of CpG-proB cells-treated mice, we analysed cytokine production by these cells (Fig. 5a,b) in a comprehensive manner, because B cells primarily inhibit immunity through cytokine production^5^,^10^. Remarkably, freshly prepared CpG-proB cells did not produce IL-10 (Fig. 5a,b) or IL-35 (Fig. 5b), but produced abundant amounts of IFN-γ, with levels about 30 times higher than those detected for the other cytokines, following stimulation for 5 h with PMA plus ionomycin (Fig. 5a). Expression of IFN-γ by these cells was also confirmed by flow cytometry (Fig. 5b) and was not significantly different in WT and IL-10-deficient CpG-proBs (Supplementary Fig. 6). Moreover, the WT CD45.1^+^ B-cell progeny recovered from reactive LN and from the spinal cord still produced IFN-γ, although less in spinal cord than in LN, as measured by qRT–PCR analysis (Fig. 5c) and intracellular flow cytometry (Supplementary Fig. 7). Since IFN-γ has protective functions in EAE^27^,^28^, this prompted us to investigate whether IFN-γ played a role in the protective function of CpG-proB cells against EAE.

We tested the role of IFN-γ using CpG-proBs prepared from IFN-γ-deficient mice. IFN-γ-deficient CpG-proB cells did not protect recipient mice against EAE on adoptive transfer (Fig. 5d). Moreover, although the cells derived from IFN-γ-deficient CpG-proBs actually reached the spinal cord, where they released as much IL-10 as the WT progeny (Supplementary Fig. 7), they also produced there fivefold more GM-CSF than the WT progeny. They failed to reduce the encephalitogenic cytokines production by CD4^+^ T cells and to enhance IL-10-producing CD4^+^ and B220^+^ cells in spinal cord (Supplementary Fig. 8). Similarly, they failed to drive an accumulation of CD4^+^ T cells in reactive LN of recipient mice, in contrast to WT CpG-proBs (Fig. 5e). This suggested to us that IFN-γ produced by donor-derived B cells contributed to protection from disease not only by limiting encephalitogenic cytokine production within the spinal cord, but also by retaining encephalitogenic T cells in reactive LN, thereby preventing their accumulation in the CNS.

To characterize how IFN-γ produced by donor-derived B cells controlled T-cell accumulation in reactive LN, we examined its effect on expression by T cells of the chemokine receptor CCR7 known to control T-cell localization within LN^29^, and to be implicated in neuro-inflammation^30^,^31^. CCR7 expression was reduced in LN CD4^+^ and CD8^+^ T cells of the CpG-proB-treated group relative to controls (Fig. 5f,g). Remarkably, CpG-proBs derived from IFN-γ-deficient mice, barely affected CCR7 staining on CD4^+^ T cells (Fig. 5f,g), contrasting with those derived from IL-10-deficient mice that were as efficient as WT CpG-proBs at retaining CD4^+^ T cells within LNs (Supplementary Fig. 9). When we performed a short acid wash (0.2 M acetic acid, 0.5 mM NaCl), known to eliminate bound ligands from their receptor^32^, before CCR7 staining, the reduction in CCR7 staining in LN T cells from CpG-proB recipients relative to controls was no longer observed (Fig. 5h,i). This suggested that increased local chemokine concentrations in LNs of CpG-proB recipients bound to CCR7 and prevented antibody binding, as shown by Britschgi *et al.*^33^. Therefore, the IFN-γ-dependent release of CCR7 ligands by CpG-proBs may play a role in inducing the retention of T cells in reactive LN in recipient mice.

### Role of CCL19 in T-cell entrapment and disease protection

**Figure 6:**
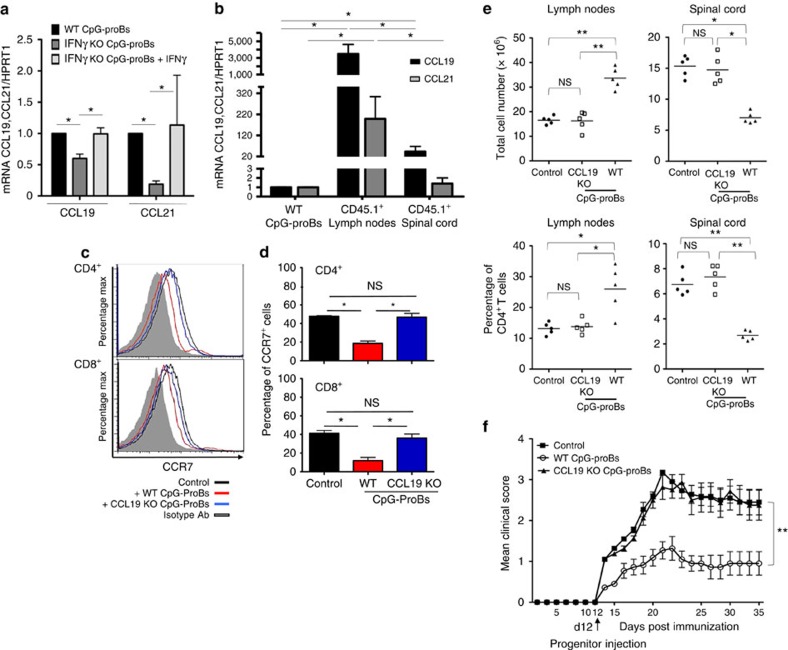
Role of CCL19 in the protective properties of CpG-proBs. (**a**) CCL19 and CCL21 mRNA levels relative to HPRT1 in WT or IFN-γ^−/−^ CpG-proB cells isolated from BM cells stimulated with CpG-B or CpG-B+IFN-γ for 18 h followed by PMA+ionomycin for 5 h. Data are expressed as means±s.e.m. of three experiments. *P<0.05 by the non-parametric Mann–Whitney's *t*-test. (**b**) CCL19 and CCL21 mRNA levels were assessed as in **a** in the progeny of WT CD45.1^+^ CpG-proBs recovered in reactive LN and spinal cord of CD45.2^+^ recipients 6 days after adoptive transfer of CD45.1^+^ CpG-proBs, and compared with levels in WT CpG-proBs. Data are expressed as mean±s.e.m. from 3–5 experiments. *P<0.05 (Students'*t*-test). (**c**,**d**) CCR7 expression analysis in CD4^+^ and CD8^+^ T cells from reactive LN of control mice (black line) and mice transferred with WT-CpG-proBs (red line) or CCL19-KO CpG-proBs (blue line), after staining with a specific anti-CCR7 antibody (open histograms) or with an isotype control (filled histogram) of intact and permeabilized cells. A representative experiment is shown in **c** and the mean±s.e.m. out of three experiments in **d**. (**e**) Control mice or mice injected at day 12 with CpG-proBs derived from WT or CCL19-deficient donors were killed on day 21 of the disease and reactive LN and spinal cord cells counted. Shown are total cell counts and percentage of CD4^+^ T cells recovered per organ, *P<0.05, **P<0.005 (Students' *t*-test). (**f**) EAE clinical scores (mean±s.e.m.) in control mice (*n*=10) or mice injected either with WT (*n*=10) or CCL19-deficient CpG-proBs (*n*=10). ***P<0.001, between controls or CCL19^−/−^ and WT CpG-proB recipients, non significant between controls and CCL19^−/−^ CpG-proB-treated mice, using the two-way repeated measures ANOVA test.

The protection afforded by B-cell-derived IFN-γ identifies a new mechanism of B-cell-mediated regulation in EAE. We sought to gain further insight into this mechanism. Given that IFN-γ can modulate chemokine expression^27^, we evaluated whether CpG-proB cells and/or their progeny themselves might be a source of CCL19 and/or CCL21 via a mechanism controlled by IFN-γ. To this end, we first quantified *Ccl19* and *Ccl21* mRNA in WT CpG-proBs, IFN-γ-deficient CpG-proBs, and IFN-γ-deficient CpG-proBs cultured in presence of IFN-γ. We found that WT CpG-proB cells produced both CCL19 and CCL21 (Fig. 6a). The production of these chemokines was reduced in IFN-γ-deficient CpG-proBs, and this defect could be erased by exogenous addition of IFN-γ (Fig. 6a). Remarkably, in LN, the mature B-cell progeny generated from CpG-proBs in recipients expressed markedly higher levels of *Ccl21*, and even more *Ccl19* than they do in spinal cord and both expressions were higher than in the originally administered CpG-proB cells (Fig. 6b). CCL19 has been shown to induce CCR7 internalization more efficiently than CCL21 (ref. 33). In addition, CD4^+^ and CD8^+^ LN T cells from mice that received WT but not *Ccl19*-deficient CpG-proBs displayed reduced cell surface expression of CCR7 (Fig. 6c,d).

Moreover, CpG-proBs sorted from *Ccl19*-deficient mice neither restrained immune cells in reactive LN (Fig. 6e), nor provided protection against EAE (Fig. 6f), confirming that CCL19 production by CpG-proB cells was essential for T-cell sequestration inside the LN and protection against disease. From these data, we conclude that CCL19 produced by CpG-proB-derived B cells in response to autocrine IFN-γ induces sequestration of T cells via CCR7 in the reactive LN, subsequently limiting their accumulation in the CNS and improving the disease course.

## Discussion

In adults, immature B cells develop in BM and then seed secondary lymphoid organs, where they can acquire diverse effector functions depending on their mode of activation. Here, we show that signals perceived by immature B cells in a BCR-independent manner can determine their subsequent effector functions as mature B cells. Indeed, we demonstrate that a transient stimulation of BM cells via TLR-9 is sufficient to divert the differentiation of pro-B precursors towards suppressive B cells. In brief, adoptive transfer of as little as 60,000 pro-B cells recovered from BM cultures shortly stimulated with CpG-B or from the BM of CpG-injected mice markedly reduced the severity of EAE in recipient mice, while unstimulated pro-B cells had no effect even though they encountered the same inflammatory environment in recipient mice. Donor-derived mature B cells mediated this protection because the latter depended on *RAG* expression.

The protection from disease mediated by the transferred CpG-proB cells involved two complementary mechanisms, one limiting local inflammation in CNS through IL-10 production, and the other restraining encephalitogenic T cells in reactive LN. The latter was achieved through LN retention of T cells, which was mediated by increased CCL19 production by CpG-proB-derived B cells under IFN-γ autocrine control. None of these effects were observed with control unstimulated pro-B cells. These data demonstrate that transient changes in the BM environment of pro-B cells can markedly influence their subsequent effector functions as mature B cells in periphery, favouring their anti-inflammatory activities.

The production of IL-10 is a key mechanism, by which B cells can suppress autoimmune diseases^4^,^5^,^6^,^11^. CpG-proBs did not produce IL-10 themselves, but their mature progeny became IL-10-competent strictly in spinal cord of recipient mice. Although CpG-proBs from IL-10-deficient mice normally retained immune cells in reactive LN, this was insufficient to provide protection against EAE since at the onset of clinical signs, pathogenic cells have already reached the CNS and triggered neuro-inflammation. We propose that IL-10 produced by donor-derived B cells is particularly important in the CNS in our model. In addition, the prevalence of IL-10 over GM-CSF production in the spinal cord by the progeny of CpG-proBs may be essential to control neuro-inflammation, as shown in Supplementary Fig. 7. The enhanced production of IL-10 by host CD4^+^ T cells and B220^+^ B cells in CNS of mice treated with WT CpG-proB might further help to control local inflammation in the target organ. In addition, mice treated with WT CpG-proB cells displayed an increased expansion of Tregs, suggesting that host Tregs are also involved in the protective effect of the transferred B cells, in line with results from other studies^15^,^19^,^34^,^35^.

An important finding of this study is the identification of a novel mechanism of B-cell-mediated immune regulation involving IFN-γ production by B cells. Looking for other non IL-10 possible mediators for the protective effects of CpG-proBs against EAE, we found that both CpG-proBs and their LN but not spinal cord progeny displayed a massive production of IFN-γ, a cytokine considered pro-inflammatory but that has also been credited with possible regulatory functions. In EAE, both IFNγ^−/−^ and IFNγR^−/−^ mice suffer from a more severe disease in both chronic and Remitting-Relapsing-EAE models^27^,^28^. The capacity to produce IFN-γ has been reported for B cells at different levels of maturation and in various activation conditions. Thus BM immature B cells constitutively produce IFN-γ at low levels^36^,^37^,^38^, whereas mature follicular and marginal zone B cells produce massive amounts of IFN-γ on TLR-activation that condition the Th1 response to *Salmonella enterica* infection^39^. Co-culture with Th1 cells emerging in pathogen-infected animals give rise to so-called Be1 effector B cells producing large amounts of IFN-γ that subsequently polarize T cells towards a type 1 immune response^40^. Bao *et al.*^41^ recently reported that innate B cells with a CD11a^hi^ CD16/32^hi^ phenotype that emerged from follicular B cells on bacterial and viral infections, produced as much IFN-γ as NK cells when re-stimulated *ex vivo* with anti-CD40. Their provision of IFN-γ was required for protection against *L. Monocytogenes* infection. Conversely, Olalekan *et al.*^42^ reported that IFN-γ production by B cells was essential for the development of autoimmune experimental arthritis. Therefore, IFN-γ production by CpG-proBs as well as other described B cells have functional, either protective or deleterious, roles and may link innate and adaptive immune responses.

Since PD-L1^hi^ mature splenic B cells were recently shown to provide protection against EAE^12^ and PD-L1 can be induced by IFN-γ, we investigated the possible role of PD-L1 in CpG-proB-induced protection. There was no correlation between the level of PD-L1 expression on WT versus IL-10 and IFN-γ-deficient CpG-proBs and their relative protective capacity against EAE (Supplementary Fig. 10a). Furthermore, CpG-proBs isolated from PD-L1-deficient C57BL/6 donor mice showed only a slight (non significant) reduction in their protective effect against EAE (Supplementary Fig. 10b). Therefore, PD-L1 is not playing a significant role in disease protection against EAE by CpG-proBs.

Our data indicate that the protective effect mediated by B-cell-derived IFN-γ operates at least in part by sequestering CD4^+^ T cells in the reactive LN. Many presently available treatments of MS aim at preventing T cells from entering the CNS. Several chemokine receptors and their ligands have been shown to take part in the process of neuroinflammation, including CCR7 whose ligands CCL19 and CCL21 are produced by endothelial cells at the inflamed BBB in mice and men^43^,^44^. CCR7^−/−^ or *plt/plt* mice that lack CCL19 and CCL21 are resistant to EAE^45^, demonstrating the requirement of CCL19/CCR7 interactions for the development of EAE. When expressed in peripheral tissues, CCL19 and CCL21 are able to mediate cell recruitment *in vivo* of both naive and recently activated T cells^46^. CpG-proB recipients showed downregulated staining for CCR7 at the surface of both CD4^+^ and CD8^+^ LN T cells. We found that IFN-γ affected CCR7 expression on T cells indirectly by enhancing the production by CpG-proB cells of both CCR7 ligands, CCL19 and CCL21. Once in the LN, the B-cell progeny produced both chemokines at far higher rates than the progenitors they derived from. The observation that similar levels of cell surface CCR7 were recovered once ligands were eliminated by an acid wash suggested that high-receptor occupancy by CCL19 or CCL21 was taking place in T cells from CpG-proB recipients, anchoring T cells within LN. Britschgi *et al.*^33^ have demonstrated that CCR7 levels and occupancy reflect the amount of CCL19 present in the environment and that high levels of CCL19 reduce CCR7 staining by the same antibody we used. Similarly, increased levels of the CCR7 ligands in inflamed tissues have been reported to reduce CCR7 detection and to induce locally T-cell retention^47^. Thus, high occupancy of CCR7 by its ligands and particularly CCL19 occurs in LN of CpG-proB recipients and impedes leucocyte migration. The particular anchoring role of CCL19 produced by the B-cell progeny was confirmed by the findings that CpG-proB cells isolated from CCL19-deficient mice did not reduce surface CCR7 expression measured without acidic washing procedure and concomitantly lost their capacity to sequester T cells within reactive LN, and to protect against EAE disease. Importantly, the markedly (3,000-fold) enhanced production of CCL19 in LN rather than spinal cord progeny of CpG-proBs strongly supports a major role of this protective mechanism in the reactive LN to keep T cells away from the CNS. Notably, we did not observe significant reduction of S1P1R expression of LN-trapped T cells in CpG-proB recipients relative to control mice with EAE. Therefore, different from FTY720 (ref. 48) which targets the LN egress signal, CpG-proBs promote entrapment of pathogenic T cells inside secondary lymphoid organs by targeting instead the retention signal.

**Figure 7:**
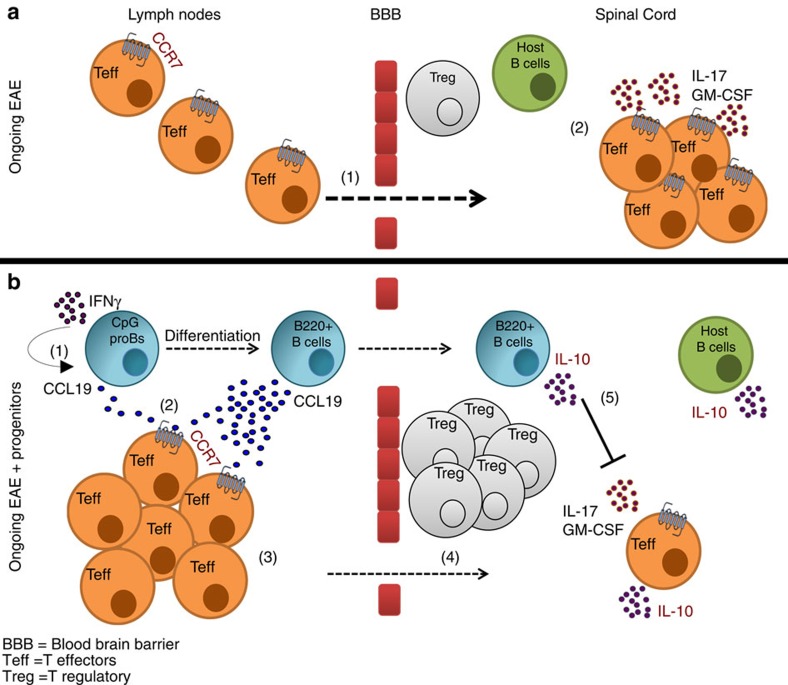
Protection against EAE by CpG-proBs relies on cooperative action of their LN and spinal cord B-cell progeny. (**a**) At the onset of clinical signs of EAE in untreated mice, autoreactive T cells migrate to the spinal cord (1) and release pathogenic cytokines IL-17 and GM-CSF (2). (**b**) In mice protected against EAE by adoptive transfer of only 60,000 CpG-proBs per recipient, the IFN-γ-dependent release by CpG-proB's progeny of CCL19 (1–2) anchors T cells via CCR7 (3), leading to effector T cells (Teff) accumulation in reactive LN. As a result, fewer T cells reach the spinal cord (4). In the spinal cord, the CpG-proB's progeny releases IL-10 (5), that reduces IL-17 and GM-CSF production by T cells, enhances Foxp3^+^ Treg accumulation and triggers IL-10 release by host B cells.

In summary (Fig. 7), the powerful protective effect of CpG-proBs against EAE is due to the capacity of their progeny to anchor T cells within the LNs particularly by releasing CCL19, thereby retaining them away from the spinal cord. This process is indispensable, but not sufficient to confer protection against EAE, at disease onset when T cells have already migrated into the CNS. The B220^+^CD11b^+^CD1d^+^CD5^+^ B-cell progeny of injected CpG-proBs must further release IL-10 within the spinal cord, which is ultimately required for switching the host cytokine profile from inflammatory to immunoregulatory. These molecular mechanisms were obtained using progenitors in which each individual mediator was deficient at a time. BM chimera exhibiting concomitant deficiencies in all the mentioned molecular mediators may be necessary to provide full demonstration of their roles in the protection afforded by CpG-proBs. CpG-proBs are, to our knowledge, the most efficient B-cell subset so far reported to be able, at only 60,000 cells per recipient, to stably inhibit ongoing encephalomyelitis. These properties of CpG-proBs therefore open interesting perspectives for cell therapy of autoimmune diseases. In addition, these data shed light on the importance of the BCR-independent signals that B-cell progenitors receive in the BM environment to develop into mature B cells with regulatory rather than inflammatory functions.

## Methods

### Mice

WT CD45.2^+^ C57BL/6J mice were obtained from Janvier Laboratories (Le Genest Saint Isle, France). Congenic CD45.1^+^, MyD88^−/−^, IFN-γ^−/−^, Rag2^−/−^ C57BL/6J, all backcrossed for at least ten generations, were bred in our animal facility under specific pathogen-free conditions. CCL19^−/−^ C57BL/6J mice were raised at the Department of Biochemistry at the University of Lausanne, IL-10^−/−^ C57BL/6J mice at the DRFZ, Berlin and PD-L1^−/−^ C57BL/6J mice^49^ at Trinity Biomedical Sciences Institute, Trinity College Dublin. Knock-out animals and their WT controls from the same origin were systematically used as donor mice for CpG-proBs. Female 12-week-old C57BL/6J mice were used as a model for MOG_35–55_-induced EAE and received intravenous progenitor cell transfers at day-12 post-immunization.

### EAE induction

Active EAE was induced in 12-week-old female mice by s.c. immunization at two sites, upper and lower back, with 200 μg MOG_35–55_ peptide emulsified in CFA containing 400 μg heat-killed *Mycobacterium tuberculosis* H37Ra (Hooke Laboratories, Lawrence, MA, USA), on day 0. In addition, mice received 200 ng pertussis toxin (Hooke Laboratories) i.p. in 0.1 ml per mouse on days 0 and 1. Clinical signs of EAE were assessed daily with a 0- to 5-point scoring system defined as follows: 0, no obvious changes in motor function compared with non-immunized mice; 0.5, tip of tail is limp; 1, limp tail; 1.5, limp tail and hind leg inhibition; 2, limp tail and weakness of hind legs; 2.5, limp tail and dragging of hind legs; 3, limp tail and complete paralysis of hind legs or paralysis of one front and one hind leg; 3.5, limp tail and complete paralysis of hind legs that are together on one side of body; 4, limp tail, complete hind leg and partial front leg paralysis; mouse is still minimally moving and appears feeding; 4.5, complete hind leg and partial front leg paralysis; no movement around the cage, mouse is not alert; 5, death or severe paralysis. Mice with score ≥4 for two consecutive days and mice with score 5 were killed.

### Sorting of TLR-activated BM progenitors

BM cells removed from tibiae and femurs of 8–12-week-old C57BL/6J mice were incubated in RPMI-1640 medium (PAA) supplemented with 10% (vol/vol) fetal calf serum and 1% antibiotics (penicillin and streptomycin) for 18 h with 1 μg ml^−1^ of the oligodeoxynucleotides CpG 1,585 (CpG-A; InvivoGen), CpG 1,668 (CpG-B; Eurogentec, Angers, France) or with the respective agonists of TLR-1–9, supplied in a commercial kit (InvivoGen, Toulouse, France), including: Pam3CSK4 (0.5 μg ml^−1^), FSLI (1 μg ml^−1^), HKLM (2 × 10^6^ cells ml^−1^), Poly I:C (HMW; 5 μg ml^−1^), Poly I:C (LMW; 5 μg ml^−1^), LPS-EK standard (1 μg ml^−1^), FLA- ST standard (1 μg ml^−1^) and ssRNA40/LyoVec (2 μg ml^−1^) as well as CpG 1,826 (CpG-B; 1 μg ml^−1^).

c-kit^+^ BM cells were sorted by immuno-magnetic separation using a RoboSep automaton (StemCell Technologies, Grenoble, France). Sorted cells were further stained with appropriate fluorochrome-conjugated mAbs against Sca-1, B220, PDCA-1, IgM and electronically sorted as a small-size, c-kit^low^Sca-1^low^B220^+^PDCA-1^−^IgM^−^ cell subset using a FACS-Aria I (BD Biosciences, Le Pont de Claix, France).

### Isolation of immune cells from the spinal cord

Spinal cords isolated from control and CpG-proB-recipient mice were incubated for 30 min in digestion buffer of DNAse and liberase (27 WU ml^−1^) in PBS 1 × at 37 °C, mixing every 5 min. EDTA (100 mM, 500 μl) was added for 1 min to end the digestion. The cells were passed through a cell strainer, using a syringe plunger (back side) to smash the tissue. Cells were then resuspended in 3–5 ml of 40% Percoll underlayed with the same volume of 70% Percoll (in PBS) and centrifuged for 35 min at 1,300 g (2,800 r.p.m.) without brakes to form a smooth interface. Cells were collected with a Pasteur pipette and diluted 10 times with complete medium RPMI 10% SVF. After centrifugation cells were resuspended in 2–3 ml of complete medium and further stained with appropriately labelled mAbs.

### Staining of cells for flow cytometry analysis

To block non-specific Fc receptor binding, cells were pre-incubated for 10 min at room temperature with FcR blocker 2.4G2 mAb. Cells were then stained with appropriately labelled mAbs against CD4, B220, CD21, CD23, CD24, IgM, IgD, IgG, CD1d, CD5, CD43, CD93, PDCA-1, CD8 (eBioscience, Paris, France), CD19, CD127, CD25, CD11b, CD11c, c-Kit (CD117), Sca-1 (anti-Ly6A/E), CCR7 (clone 4B12), CD25, CD45.1 (BD Biosciences) and PDCA-1 (Miltenyi Biotec, Paris, France). Nuclear Foxp3 expression was measured by FACS analysis as per the manufacturer's instructions (eBioscience). Pax5 expression was measured in B-cell progenitors permeabilized with the same buffer as for Foxp3, using an anti-Pax5 antibody from eBioscience. Intracytoplasmic expression of cytokines was assessed after a 5-h stimulation with PMA (10 ng ml^−1^) plus ionomycin (500 ng ml^−1^) in the presence of Brefeldin A (20 μg ml^−1^), followed by fixation/permeabilization with PFA/saponin and subsequent staining with specific antibodies including APC-labelled anti-IL-23p19, APC-labelled anti-IL-12p40, APC-labelled anti-IL-10, PE-labelled anti–IFN-γ, APC-labelled anti-IL-17 (all from BD Biosciences), PE-labelled anti-GM-CSF (from eBioscience), Per-CP-labelled anti-human/mouse IL-12/IL-35 p35 (from RnD Systems) or isotype controls (from BD Biosciences and eBioscience). Membrane and intracellular antigen expression was analysed in a FACS Canto II cytometer (BD Biosciences) using FlowJo software (Treestar, Ashland, OR, USA).

### Cytokine assays

Control and CpG-ProB-recipient mice were killed at the peak of the disease, 18–21 days after immunization. Cytokines were measured in supernatants of recovered LN cells from control or CpG-ProB-recipient mice, re-stimulated with the MOG_35–55_ peptide for 3 days followed by 5-h stimulation with PMA+ionomycin. For determination of spinal cord cytokines, isolation of spinal cord cells was followed by incubation at 37 °C/5% CO_2_ in the presence of Golgi-stop (+PMA/ionomycin) for 3 h. Th1/Th2/Th17 cytokines and GM-CSF were measured by multiplex ELISA using Flow Cytomix analyte detection reagents from eBioscience.

### Isolation of mRNA and real-time qRT–PCR

Total RNA was prepared using RNAqueous-4PCR (Ambion, Life Technologies, St Aubin, France). Reverse-transcription was performed with high-capacity cDNA reverse-transcription kits (Applied Biosystems, Life Technologies). Resulting cDNA was amplified in triplicates by the SYBR-Green PCR assay. PCR reactions were incubated for 2 min at 50 °C and for 10 min at 95 °C, followed by 40 amplification cycles with 1 min annealing/extension at 60 °C and 15-s denaturation at 95 °C. Quantitative real-time PCR of mouse CCL19 and CCL21 was performed by the comparative threshold cycle (ΔΔC_T_) method and normalized to mouse HPRT1 using AB 7,900 HT real-time PCR system (Applied Biosystems). The primer sequences used for CCL19 identify functional CCL19 (ref. 50). Primer sequences were as described^50^: CCL19 forward: 5′-CTGCCTCAGATTATCTCGCAT-3′, CCL19 reverse: 5′-GTCTTCCGCATCATTAGCAC-3′; CCL21 forward: 5′-ATCCCGGCAATCCTGTTCTC-3′, CCL21 reverse: 5′-GGTTCTGCACCCAGCCTTC-3′; HPRT1 forward: 5′-CCTTCACCAATGACTCCTATGAC-3′, HPRT1 reverse: 5′-CAAGTTTACAGCCAAGATTCAC-3′.

### Study approval

All mouse procedures were approved by the Paris Descartes University Animal Experimentation and Ethics Committee. Sample sizes were chosen to ensure reproducibility of the experiments and according to the 3Rs of animal ethics regulation.

### Statistics

Non-parametric Mann–Whitney's *t*-test was used to compare values between the two groups. Disease curves were analysed using two-way repeated measures ANOVA test.

### Data availability

All relevant data are available from the authors.

## Additional information

**How to cite this article:** Korniotis, S. *et al.* Treatment of ongoing autoimmune encephalomyelitis with activated B-cell progenitors maturing into regulatory B cells. *Nat. Commun.* 7:12134 doi: 10.1038/ncomms12134 (2016).

## Supplementary Material

Supplementary Information Supplementary Figures 1-10

## Figures and Tables

**Table 1:** Adoptive transfer of CpG-proB cells but not of control pro-B cells inhibits ongoing EAE.

**Group**	**Mice**	**Mean maximum score**±**s.e.m.**	**Day of onset**±**s.e.m.**	**Disease incidence**
Control	30	3.51±0.89	13.56±1.40	100%
WT CpG-proBs	30	1.56±1.26**	14.50±2.22	76.7%*
Control ProBs	20	3.57±0.92	13.30±2.22	100%
PBS-proBs	4	3.50±0.05	12.00±0.00	100%

Clinical parameters from data in Fig. 1e, including the maximum clinical score (mean±s.e.m.) of each treatment group and the day of disease onset (mean±s.e.m.) among mice with EAE as well as the disease incidence over the entire observation period (35 days). Control mice received PBS injection instead of progenitor injection. Significant differences between CpG-proB recipients versus control mice are indicated; *P<0.05, **P<0.005, assessed by non-parametric Mann–Whitney's *t*-test.

## References

[b1] Lund F. E. & Randall T. D. Effector and regulatory B cells: modulators of CD4^+^ T cell immunity. Nat. Rev. Immunol. 10, 236–247 (2010). 20224569 10.1038/nri2729 PMC3038334

[b2] Hsue P. Y. *et al.* Depletion of B-cells with rituximab improves endothelial function and reduces inflammation among individuals with rheumatoid arthritis. J. Am. Heart Assoc. 3, e001267 (2014). 25336464 10.1161/JAHA.114.001267 PMC4323827

[b3] Lehmann-Horn K., Kronsbein H. C. & Weber M. S. Targeting B cells in the treatment of multiple sclerosis: recent advances and remaining challenges. Ther. Adv. Neurol. Disord. 6, 161–173 (2013). 23634189 10.1177/1756285612474333 PMC3625013

[b4] Mizoguchi A., Mizoguchi E., Takedatsu H., Blumberg R. S. & Bhan A. K. Chronic intestinal inflammatory condition generates IL-10-producing regulatory B cell subset characterized by CD1d upregulation. Immunity 16, 219–230 (2002). 11869683 10.1016/s1074-7613(02)00274-1

[b5] Fillatreau S., Sweenie C. H., McGeachy M. J., Gray D. & Anderton S. M. B cells regulate autoimmunity by provision of IL-10. Nat. Immunol. 3, 944–950 (2002). 12244307 10.1038/ni833

[b6] Mauri C., Gray D., Mushtaq N. & Londei M. Prevention of arthritis by interleukin 10-producing B cells. J. Exp. Med. 197, 489–501 (2003). 12591906 10.1084/jem.20021293 PMC2193864

[b7] Evans J. G. *et al.* Novel suppressive function of transitional 2 B cells in experimental arthritis. J. Immunol. 178, 7868–7878 (2007). 17548625 10.4049/jimmunol.178.12.7868

[b8] Yanaba K. *et al.* A regulatory B cell subset with a unique CD1dhiCD5^+^ phenotype controls T cell-dependent inflammatory responses. Immunity 28, 639–650 (2008). 18482568 10.1016/j.immuni.2008.03.017

[b9] Shimomura Y. *et al.* Regulatory role of B-1 B cells in chronic colitis. Int. Immunol. 20, 729–737 (2008). 18375938 10.1093/intimm/dxn031

[b10] Shen P. *et al.* IL-35-producing B cells are critical regulators of immunity during autoimmune and infectious diseases. Nature 507, 366–370 (2014). 24572363 10.1038/nature12979 PMC4260166

[b11] Matsumoto M. *et al.* Interleukin-10-producing plasmablasts exert regulatory function in autoimmune inflammation. Immunity 41, 1040–1051 (2014). 25484301 10.1016/j.immuni.2014.10.016

[b12] Khan A. R. *et al.* PD-L1hi B cells are critical regulators of humoral immunity. Nat. Commun. 6, 5997 (2015). 25609381 10.1038/ncomms6997

[b13] Lampropoulou V. *et al.* TLR-activated B cells suppress T cell-mediated autoimmunity. J. Immunol. 180, 4763–4773 (2008). 18354200 10.4049/jimmunol.180.7.4763

[b14] Yanaba K., Bouaziz J. D., Matsushita T., Tsubata T. & Tedder T. F. The development and function of regulatory B cells expressing IL-10 (B10 cells) requires antigen receptor diversity and TLR signals. J. Immunol. 182, 7459–7472 (2009). 19494269 10.4049/jimmunol.0900270 PMC3733128

[b15] Mauri C. & Bosma A. Immune regulatory function of B cells. Annu. Rev. Immunol. 30, 221–241 (2012). 22224776 10.1146/annurev-immunol-020711-074934

[b16] Baba Y., Matsumoto M. & Kurosaki T. Calcium signaling in B cells: regulation of cytosolic Ca2^+^ increase and its sensor molecules, STIM1 and STIM2. Mol. Immunol. 62, 339–343 (2014). 24246800 10.1016/j.molimm.2013.10.006

[b17] Mann M. K., Maresz K., Shriver L. P., Tan Y. & Dittel B. N. B cell regulation of CD4^+^CD25^+^ T regulatory cells and IL-10 via B7 is essential for recovery from experimental autoimmune encephalomyelitis. J. Immunol. 178, 3447–3456 (2007). 17339439 10.4049/jimmunol.178.6.3447

[b18] Yoshizaki A. *et al.* Regulatory B cells control T-cell autoimmunity through IL-21-dependent cognate interactions. Nature 491, 264–268 (2012). 23064231 10.1038/nature11501 PMC3493692

[b19] Blair P. A. *et al.* Selective targeting of B cells with agonistic anti-CD40 is an efficacious strategy for the generation of induced regulatory T2-like B cells and for the suppression of lupus in MRL/lpr mice. J. Immunol. 182, 3492–3502 (2009). 19265127 10.4049/jimmunol.0803052 PMC4082659

[b20] Duddy M. *et al.* Distinct effector cytokine profiles of memory and naive human B cell subsets and implication in multiple sclerosis. J. Immunol. 178, 6092–6099 (2007). 17475834 10.4049/jimmunol.178.10.6092

[b21] Correale J. & Farez M. F. Does helminth activation of toll-like receptors modulate immune response in multiple sclerosis patients? Front. Cell. Infect. Microbiol. 2, 112 (2012). 22937527 10.3389/fcimb.2012.00112 PMC3426839

[b22] Ramgolam V. S. *et al.* B cells as a therapeutic target for IFN-beta in relapsing-remitting multiple sclerosis. J. Immunol. 186, 4518–4526 (2011). 21368231 10.4049/jimmunol.1000271

[b23] Begum-Haque S. *et al.* Augmentation of regulatory B cell activity in experimental allergic encephalomyelitis by glatiramer acetate. J. Neuroimmunol. 232, 136–144 (2011). 21111489 10.1016/j.jneuroim.2010.10.031 PMC3753076

[b24] Kala M. *et al.* B cells from glatiramer acetate-treated mice suppress experimental autoimmune encephalomyelitis. Exp. Neurol. 221, 136–145 (2010). 19879259 10.1016/j.expneurol.2009.10.015

[b25] Montandon R. *et al.* Innate pro-B-cell progenitors protect against type 1 diabetes by regulating autoimmune effector T cells. Proc. Natl Acad. Sci. USA (2013). 10.1073/pnas.1222446110 PMC3683765 23716674

[b26] Welner R. S. *et al.* Lymphoid precursors are directed to produce dendritic cells as a result of TLR9 ligation during herpes infection. Blood 112, 3753–3761 (2008). 18552210 10.1182/blood-2008-04-151506 PMC2572801

[b27] Tran E. H., Prince E. N. & Owens T. IFN-gamma shapes immune invasion of the central nervous system via regulation of chemokines. J. Immunol. 164, 2759–2768 (2000). 10679118 10.4049/jimmunol.164.5.2759

[b28] Hindinger C. *et al.* IFN-gamma signaling to astrocytes protects from autoimmune mediated neurological disability. PLoS ONE 7, e42088 (2012). 22848713 10.1371/journal.pone.0042088 PMC3407093

[b29] Pham T. H., Okada T., Matloubian M., Lo C. G. & Cyster J. G. S1P1 receptor signaling overrides retention mediated by G alpha i-coupled receptors to promote T cell egress. Immunity 28, 122–133 (2008). 18164221 10.1016/j.immuni.2007.11.017 PMC2691390

[b30] Noor S. & Wilson E. H. Role of C-C chemokine receptor type 7 and its ligands during neuroinflammation. J. Neuroinflammation 9, 77 (2012). 22533989 10.1186/1742-2094-9-77 PMC3413568

[b31] Engelhardt B. & Ransohoff R. M. Capture, crawl, cross: the T cell code to breach the blood-brain barriers. Trends. Immunol. 33, 579–589 (2012). 22926201 10.1016/j.it.2012.07.004

[b32] Kohout T. A. *et al.* Differential desensitization, receptor phosphorylation, beta-arrestin recruitment, and ERK1/2 activation by the two endogenous ligands for the CC chemokine receptor 7. J. Biol. Chem. 279, 23214–23222 (2004). 15054093 10.1074/jbc.M402125200

[b33] Britschgi M. R., Link A., Lissandrin T. K. & Luther S. A. Dynamic modulation of CCR7 expression and function on naive T lymphocytes *in vivo*. J. Immunol. 181, 7681–7688 (2008). 19017956 10.4049/jimmunol.181.11.7681

[b34] Amu S. *et al.* Regulatory B cells prevent and reverse allergic airway inflammation via FoxP3-positive T regulatory cells in a murine model. J. Allergy. Clin. Immunol. 125, 1114–1124 e1118 (2010). 20304473 10.1016/j.jaci.2010.01.018

[b35] Carter N. A. *et al.* Mice lacking endogenous IL-10-producing regulatory B cells develop exacerbated disease and present with an increased frequency of Th1/Th17 but a decrease in regulatory T cells. J. Immunol. 186, 5569–5579 (2011). 21464089 10.4049/jimmunol.1100284

[b36] Zavala F., Korniotis S. & Montandon R. Characterization and immunoregulatory properties of innate Pro-B-cell progenitors. Methods Mol. Biol. 1371, 79–88 (2016). 26530795 10.1007/978-1-4939-3139-2_5

[b37] Flaishon L. *et al.* Autocrine secretion of interferon gamma negatively regulates homing of immature B cells. J. Exp. Med. 192, 1381–1388 (2000). 11067886 10.1084/jem.192.9.1381 PMC2193359

[b38] Hart G., Flaishon L., Becker-Herman S. & Shachar I. Tight regulation of IFN-gamma transcription and secretion in immature and mature B cells by the inhibitory MHC class I receptor, Ly49G2. J. Immunol. 175, 5034–5042 (2005). 16210606 10.4049/jimmunol.175.8.5034

[b39] Barr T. A., Brown S., Mastroeni P. & Gray D. TLR and B cell receptor signals to B cells differentially program primary and memory Th1 responses to Salmonella enterica. J. Immunol. 185, 2783–2789 (2010). 20675594 10.4049/jimmunol.1001431 PMC3745605

[b40] Harris D. P. *et al.* Reciprocal regulation of polarized cytokine production by effector B and T cells. Nat. Immunol. 1, 475–482 (2000). 11101868 10.1038/82717

[b41] Bao Y. *et al.* Identification of IFN-gamma-producing innate B cells. Cell. Res. 24, 161–176 (2014). 24296781 10.1038/cr.2013.155 PMC3915900

[b42] Olalekan S. A., Cao Y., Hamel K. M. & Finnegan A. B cells expressing IFN-gamma suppress Treg-cell differentiation and promote autoimmune experimental arthritis. Eur. J. Immunol. 45, 988–998 (2015). 25645456 10.1002/eji.201445036 PMC4438566

[b43] Alt C., Laschinger M. & Engelhardt B. Functional expression of the lymphoid chemokines CCL19 (ELC) and CCL 21 (SLC) at the blood-brain barrier suggests their involvement in G-protein-dependent lymphocyte recruitment into the central nervous system during experimental autoimmune encephalomyelitis. Eur. J. Immunol. 32, 2133–2144 (2002). 12209625 10.1002/1521-4141(200208)32:8<2133::AID-IMMU2133>3.0.CO;2-W

[b44] Columba-Cabezas S., Serafini B., Ambrosini E. & Aloisi F. Lymphoid chemokines CCL19 and CCL21 are expressed in the central nervous system during experimental autoimmune encephalomyelitis: implications for the maintenance of chronic neuroinflammation. Brain. Pathol. 13, 38–51 (2003). 12580544 10.1111/j.1750-3639.2003.tb00005.x PMC8095989

[b45] Kuwabara T. *et al.* CCR7 ligands are required for development of experimental autoimmune encephalomyelitis through generating IL-23-dependent Th17 cells. J. Immunol. 183, 2513–2521 (2009). 19625643 10.4049/jimmunol.0800729

[b46] Luther S. A. *et al.* Differing activities of homeostatic chemokines CCL19, CCL21, and CXCL12 in lymphocyte and dendritic cell recruitment and lymphoid neogenesis. J. Immunol. 169, 424–433 (2002). 12077273 10.4049/jimmunol.169.1.424

[b47] McNamee E. N. *et al.* Ectopic lymphoid tissue alters the chemokine gradient, increases lymphocyte retention and exacerbates murine ileitis. Gut 62, 53–62 (2013). 22267601 10.1136/gutjnl-2011-301272 PMC3726216

[b48] Brinkmann V. *et al.* Fingolimod (FTY720): discovery and development of an oral drug to treat multiple sclerosis. Nat. Rev. Drug. Discov. 9, 883–897 (2010). 21031003 10.1038/nrd3248

[b49] Dong H. *et al.* B7-H1 determines accumulation and deletion of intrahepatic CD8^(+)^ T lymphocytes. Immunity 20, 327–336 (2004). 15030776 10.1016/s1074-7613(04)00050-0

[b50] Luther S. A., Tang H. L., Hyman P. L., Farr A. G. & Cyster J. G. Coexpression of the chemokines ELC and SLC by T zone stromal cells and deletion of the ELC gene in the plt/plt mouse. Proc. Natl Acad. Sci. USA 97, 12694–12699 (2000). 11070085 10.1073/pnas.97.23.12694 PMC18826

