# Supplementary material for: Treatment of ongoing autoimmune encephalomyelitis with activated B-cell progenitors maturing into regulatory B cells
Source: Nat Commun. 2016 Jul 11;7:12134. doi: 10.1038/ncomms12134 (PMC4942579; doi:10.1038/ncomms12134)
Supplement: Supplementary Information — Supplementary Figures 1-10 [file ncomms12134-s1.pdf]

Korniotis et al. Supplementary figures

a.

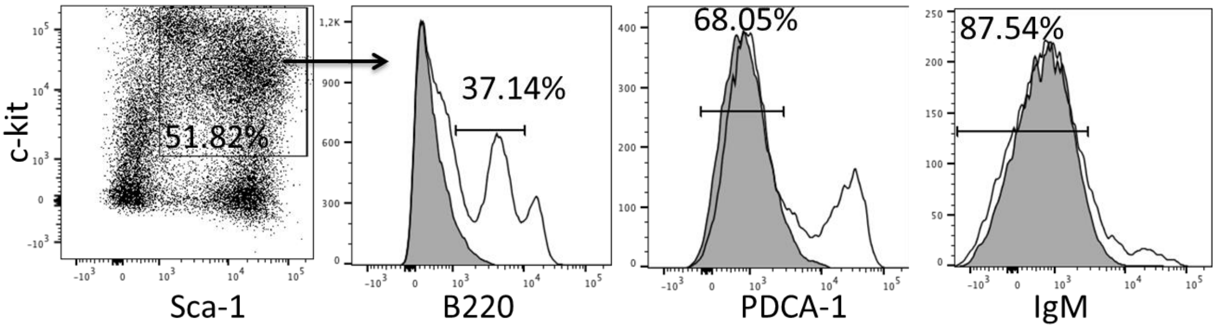

b.

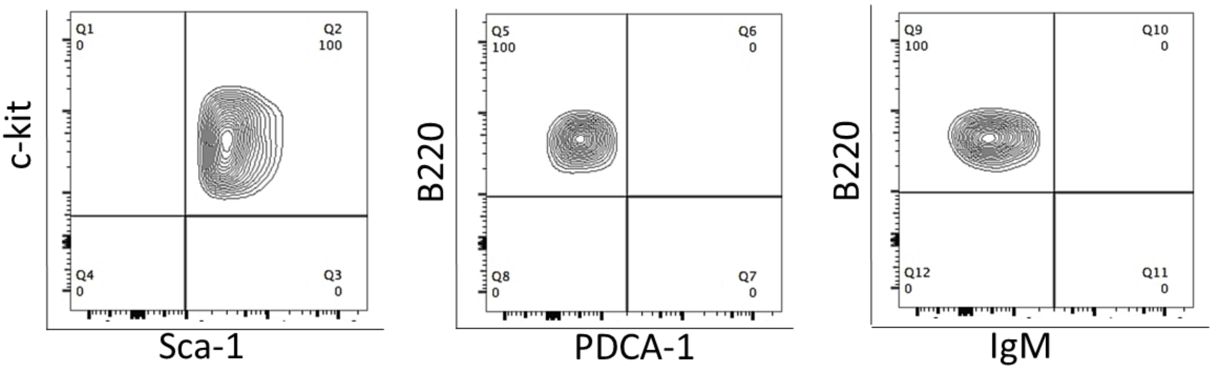

Supplementary Fig. 1. Negative controls used for the cell sorting procedure of CpG-proBs and purity assessment of the final sorted population. a. CpG-incubated bone marrow cells obtained by positive c-kit^{+} magnetic selection were sorted electronically as c-kit^{+} Sca-1^{+}B220^{+} PDCA-1^{-}IgM^{-}. Isotype controls (grey shadow) were used to assess expression of Sca-1, B220, PDCA-1 and IgM and define the sorting gates. **b**. The final cell-sorted CpG-proBs were checked for the absence of contaminating cells. FACS analysis confirms that only cells sorted as c-kit^{+}Sca-1^{+}B220^{+}PDCA-1^{-}IgM^{-} are present within the sorted population.

Korniotis et al. Supplementary figures

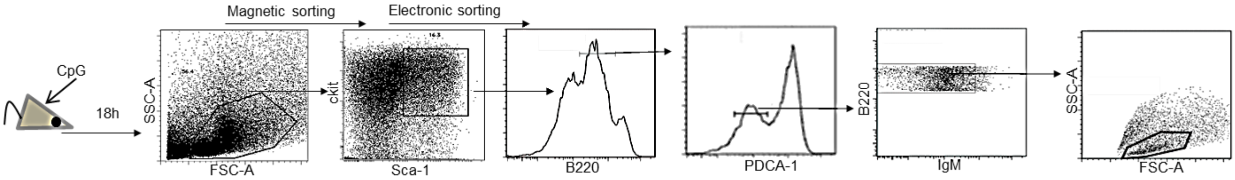

Supplementary Fig. 2. In vivo emergence and cell-sorting of CpG-proBs in CpG-treated mice. Small-size c-kit$^+$Sca-1$^+$B220$^+$PDCA$^-$IgM$^-$ cells emerge in the bone marrow of C57Bl/6J mice 18 h post injection of CpG-B (30 µg/mouse, i.p.). Magnetically sorted c-kit$^+$ bone marrow cells were further stained for Sca-1, B220, PDCA-1, IgM and electronically sorted as c-kit$^+$Sca-1$^+$B220$^+$PDCA-1$^-$IgM$^-$ cells of small size, as in Supplementary Fig.1a.

a

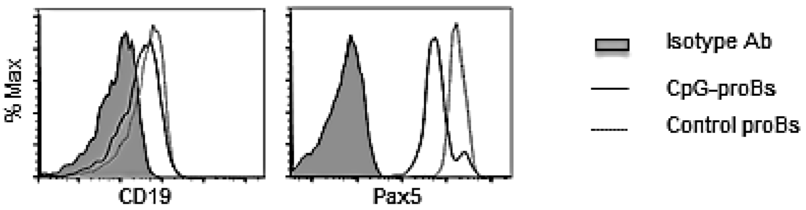

b

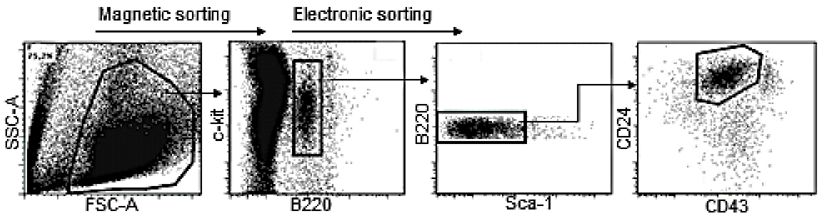

Supplementary Fig. 3. a. **CD19 and Pax5 expression by control pro-B cells and CpG-proBs.** Control pro-B cells isolated as in Fig. 1a, and in vitro prepared CpG-proBs were compared for CD19 and Pax5 expression by flow cytometry. **b**. **Cell-sorting of control bone marrow pro-B cells.** Control pro-B cells, from fresh unstimulated BM cells were magnetically sorted for c-kit^{+} cells, further stained for Sca-1, B220, CD24 and CD43 and electronically sorted as c-kit^{+}B220^{+}Sca-1^{-}CD24^{high}CD43^{high} cells.

Korniotis et al. Supplementary figures

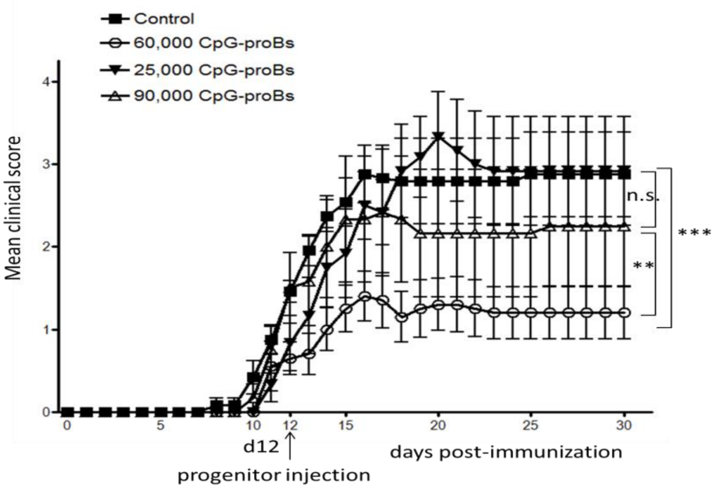

Supplementary Fig. 4. Dose-response study of CpG-proB-induced protection against EAE. The effect of 3 different doses (25,000, 60,000 and 90,000) of WT CpG-proB cells injected i.v. at day 12 in mice immunized with MOG35-55 was assessed. EAE clinical scores (mean ± s.e.m.) over 35 days of the indicated groups of mice. n = 12 mice per group for control mice, n = 6 mice per group for recipients of 90,000 and of 25,000 WT CpG-proBs, n = 10 mice per group for recipients of 60,000 WT CpG-proBs; ****P* < 0.0001 when comparing control mice injected with PBS and recipients of 60,000 WT CpG-proBs as well as mice injected with 60,000 and 25,000 CpG-proBs, by two-way repeated measures ANOVA test; ** *P* = 0.0052, between mice injected with 90,000 and 60,000 CpG-proBs, N.S. between all other groups.

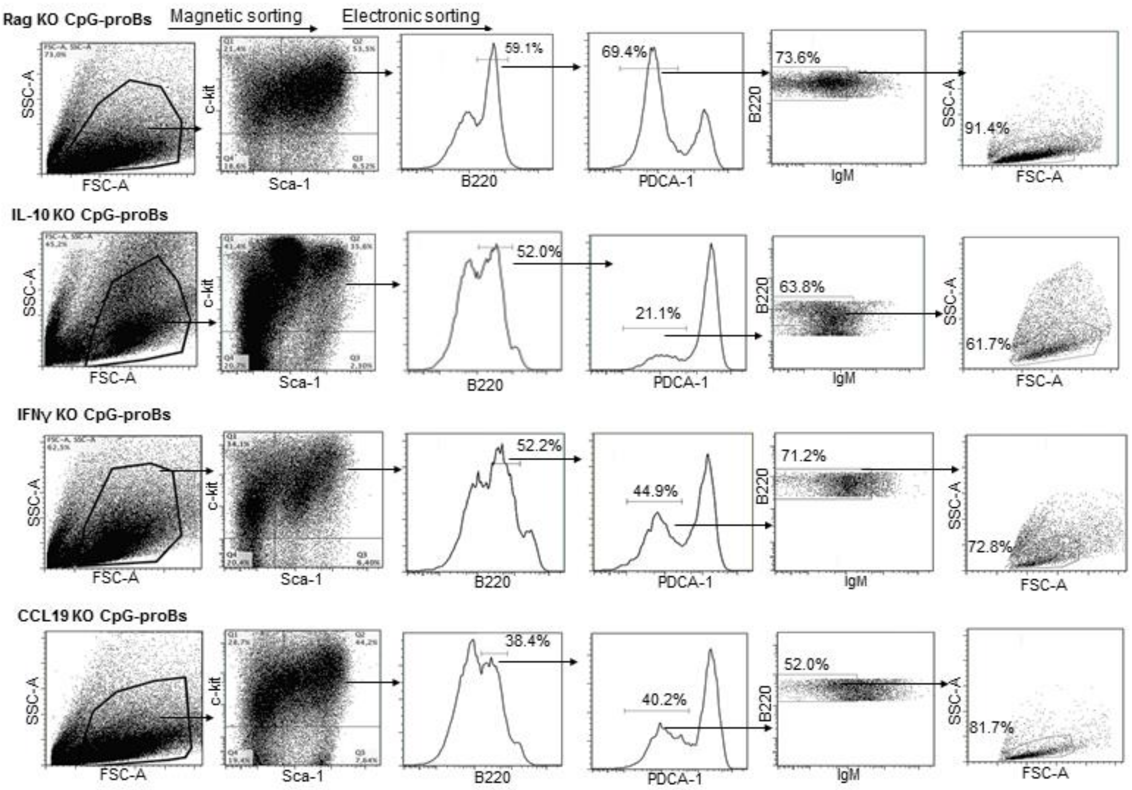

Supplementary Fig. 5. **Cell sorting procedure for the isolation of CpG-proBs from bone marrow cultures with CpG-B of *RAG*2-, IL-10-, IFN-γ- and CCL19-deficient C57BL/6 mice.**

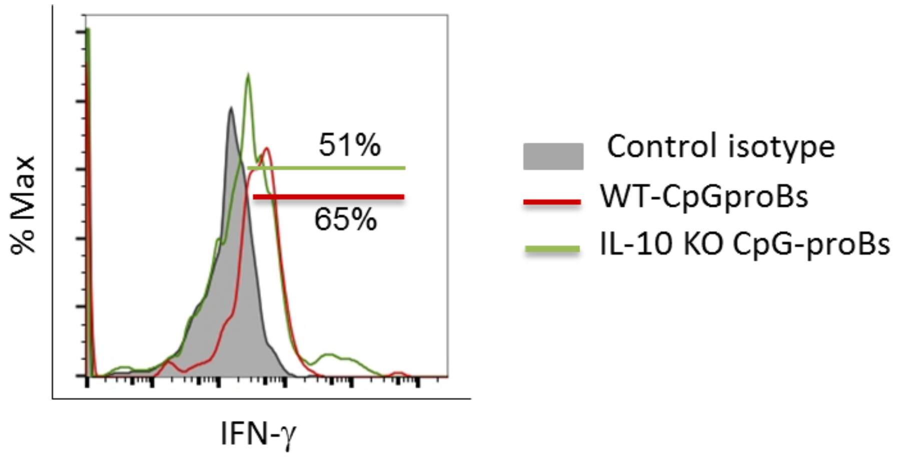

Supplementary Fig.6. IFN-γ production by WT- and IL-10-deficient CpG-proBs was determined by intracellular FACS analysis in cells permeabilized after PMA + ionomycin-stimulation for 5h.

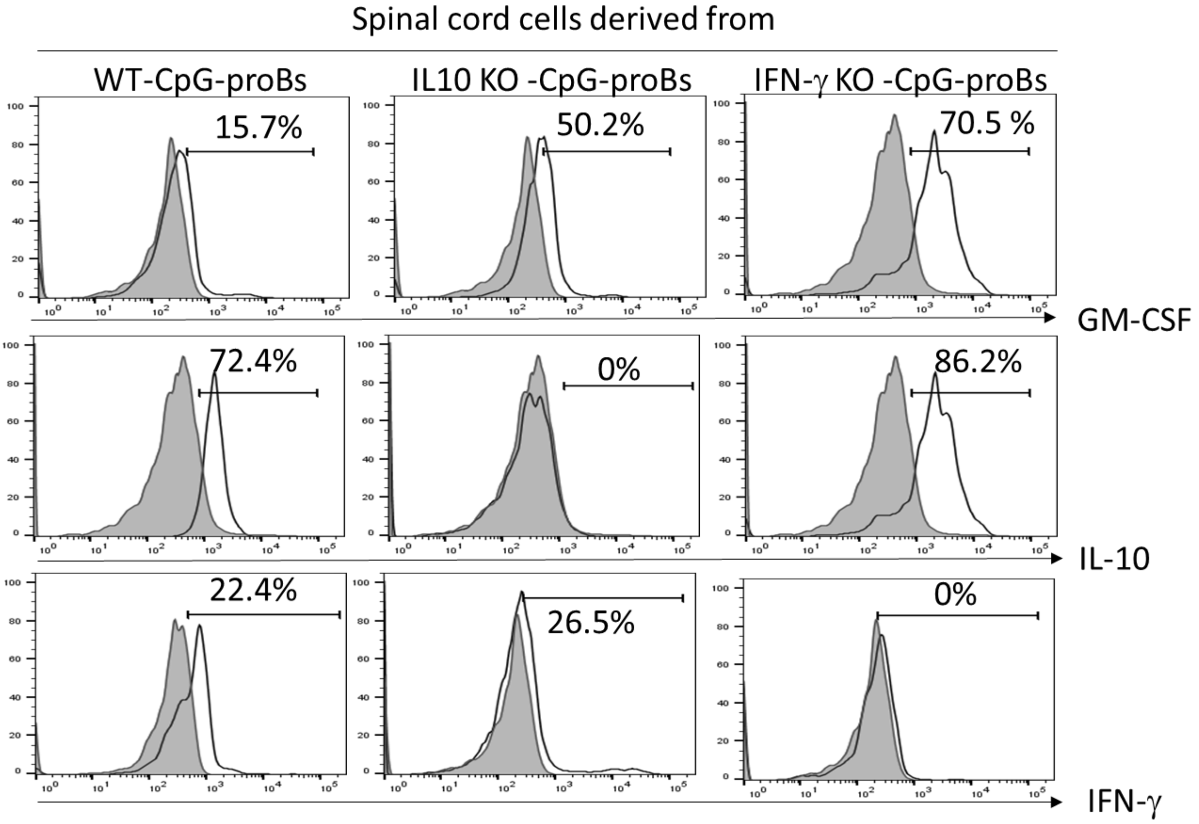

Supplementary Fig. 7. Cytokine production in the spinal cord by the progeny of WT-, IL-10- and IFN-γ-deficient CpG-proBs. Progenitors derived from WT-, IL-10- and IFN-γ-deficient CD45.2$^+$ donor mice were injected at day 12 after immunization of CD45.1$^+$ C57BL/6 recipients. At the peak of the disease, day 18-20, the cytokine production was assessed by intracellular flow cytometry within their gated CD45.2$^+$ progeny, after PMA + ionomycin stimulation of cells recovered from the spinal cord. Grey histogram, isotype control antibody; black line, specific anti-cytokine antibody. Shown is a representative experiment out of two.

Korniotis et al. Supplementary figures

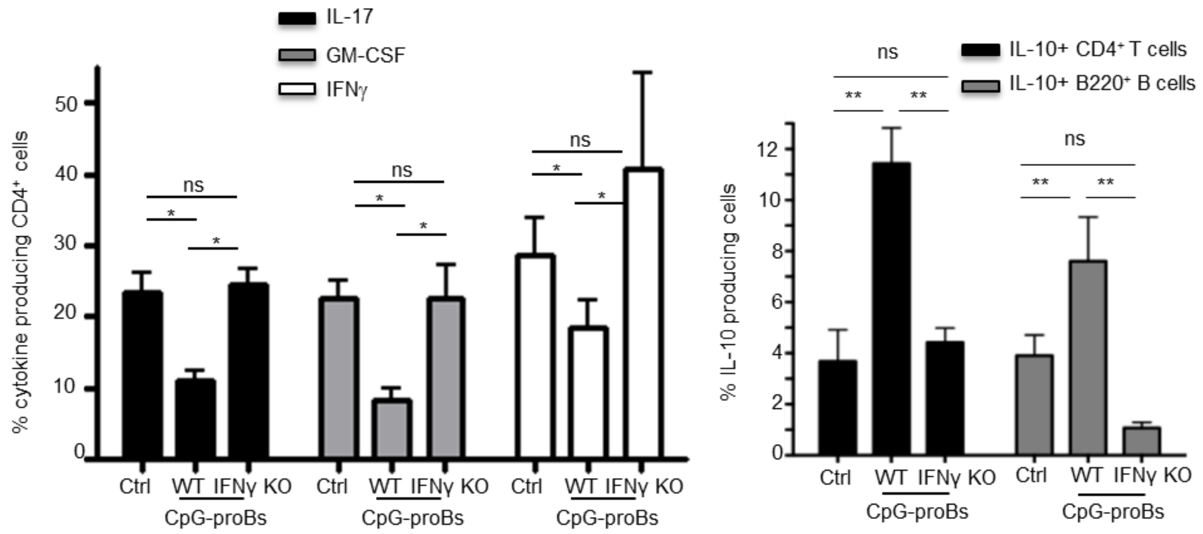

Supplementary Fig.8. Cytokine pattern in the spinal cord of controls versus recipients of WT- or IFN-γ$^{-/-}$-CpG-proBs. At the peak of the disease, day 18-20 post-immunization, the cytokine (IL-17, GM-CSF, IFN-γ, left figure; IL-10, right figure) production was assessed within gated CD4^{+} or B220^{+} cells, as indicated, after PMA + ionomycin stimulation of cells recovered from the spinal cord of control mice (n =5), versus recipients of WT- (n =5) or IFN-γ$^{-/-}$-CpG-proBs (n =4). Values are expressed as mean ± s.e.m. *, p< 0.05, **, p<0.01.

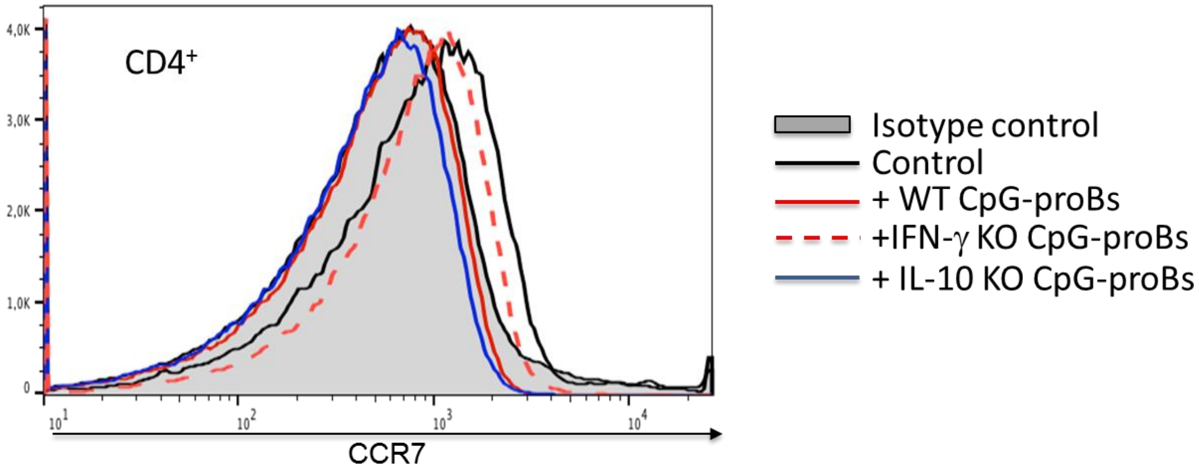

Supplementary Fig.9. Comparative effect of IFN-γ and IL-10 deficiency in CpG-proBs on the variation of T-cell CCR7 expression in T-cells of progenitor recipients. The expression of CCR7 was analyzed by flow cytometry in $CD4^+$ cells from LN of mice with EAE, either controls or transferred with WT, IFN-$\gamma^{-/-}$ or IL-$10^{-/-}$ CpG-proB cells. Shown is a representative experiment out of two.

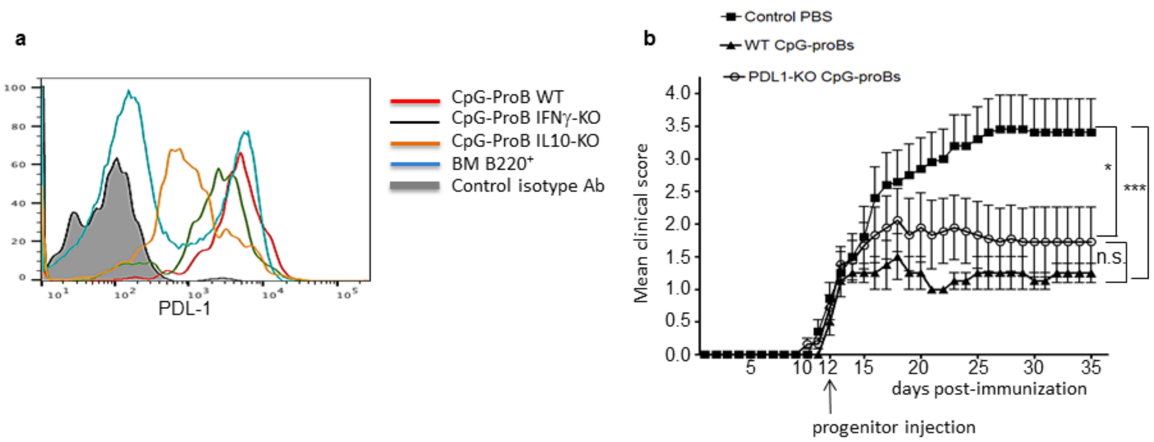

Supplementary Fig. 10. Role of PD-L1 in the protection by CpG-proBs against EAE.

a. PD-L1 expression was compared by FACS analysis, using anti-mouse PD-L1 antibody (eBiosciences) between CpG-proB cells isolated from protective WT-, or non-protective IFN-γ- and IL-10-deficient donor mice and compared to bone marrow whole B220^{+} cells. **b**. EAE mean clinical score was assessed over 35 days in control mice (n = 10) and in recipient mice of 60,000 WT- (n = 4) or PD-L1-deficient CpG-proBs (n= 9) injected at day 12 after immunization. Data are expressed as mean ± s.e.m. ****P* < 0.001 when comparing control mice injected with PBS and recipients of WT CpG-proBs; **P* = 0.0130 when comparing control mice and mice injected with PD-L1$^{-/-}$ CpG-proBs by two-way repeated measures ANOVA test. N.S., between mice injected with WT CpG-proBs and with PD-L1$^{-/-}$ CpG-proBs.
